# DelPhi Suite: New Developments and Review of Functionalities

**DOI:** 10.1002/jcc.26006

**Published:** 2019-06-25

**Authors:** Chuan Li, Zhe Jia, Arghya Chakravorty, Swagata Pahari, Yunhui Peng, Sankar Basu, Mahesh Koirala, Shailesh Kumar Panday, Marharyta Petukh, Lin Li, Emil Alexov

**Affiliations:** ^1^ Department of Mathematics West Chester University of Pennsylvania West Chester Pennsylvania 19383; ^2^ Department of Physics and Astronomy Clemson University Clemson South Carolina 29634; ^3^ Department of Biology Presbyterian College Clinton South Carolina 29325; ^4^ Department of Physics University of Texas at EI Paso Texas 79968

**Keywords:** DelPhi, Poisson‐Boltzmann equation, electrostatics, computer code parallelization, dielectric constant

## Abstract

Electrostatic potential, energies, and forces affect virtually any process in molecular biology, however, computing these quantities is a difficult task due to irregularly shaped macromolecules and the presence of water. Here, we report a new edition of the popular software package DelPhi along with describing its functionalities. The new DelPhi is a C++ object‐oriented package supporting various levels of multiprocessing and memory distribution. It is demonstrated that multiprocessing results in significant improvement of computational time. Furthermore, for computations requiring large grid size (large macromolecular assemblages), the approach of memory distribution is shown to reduce the requirement of RAM and thus permitting large‐scale modeling to be done on Linux clusters with moderate architecture. The new release comes with new features, whose functionalities and applications are described as well. © 2019 The Authors. *Journal of Computational Chemistry* published by Wiley Periodicals, Inc.

## Introduction

Electrostatics is an essential component of numerous phenomena occurring in molecular biology.^1^, ^2^ Each atom of biomolecules carries a partial charge; thus, electrostatic interactions are present at atomic level of details. Further, the electrostatic interactions, being long‐range, dominate other forces when atoms or molecules are separated at distances longer than typical bond lengths. A particular example is macromolecular binding and recognition.^3^, ^4^, ^5^, ^6^ Another example where electrostatics is the main factor is pH‐dependence of folding and binding.^7^, ^8^, ^9^, ^10^, ^11^ The list can be extended to include nonspecific ion binding,^12^, ^13^, ^14^ pKa calculations,^15^, ^16^, ^17^ and salt‐dependent effects.^18^, ^19^

However, modeling electrostatic potential and energies in systems containing macromolecules made of millions of atoms and more in the presence of water molecules and mobile ions is an extremely complicated task. Explicit solvent modeling requires significant computational resources, takes a long time, and faces questions about the convergence. Alternatives are offered by implicit solvent models such as Poisson Boltzmann (PB) and Generalized Born approaches.^20^, ^21^, ^22^, ^23^, ^24^, ^25^ Particularly, PB approach has been explored by many researchers resulting into various software such as DelPhi,^26^, ^27^ PBSA,^28^ MIBPB,^29^ APBS,^30^ and others.^31^, ^32^

Here, we report a complete renovation of DelPhi software and associated resources.^26^ The new DelPhi C++ is redesigned to utilize the object‐oriented (OO) programming technique and other unique features provided by C++ to transform the single‐model single‐solver code into a multimodel multisolver platform with diverse user interfaces, unified data encapsulation and accesses, and flexible model‐solver pairing mechanism. DelPhi C++ package integrates three implementations (regular single‐CPU, multithreaded, and multi‐CPU parallel) in one set of code, allowing users to compile one or more desired implementations with minimal effort. Numerous new features are added expanding DelPhi capabilities to model various phenomena in molecular biology. Here, we briefly outline these new features, provide examples and access the accuracy of calculations against analytical solutions at different grid resolutions. Furthermore, various methods, software, and webservers were developed utilizing DelPhi. These resources include changes of folding (SAAMBE^33^ method) and binding (SAAFEC^34^, ^35^ and SAMPDI^36^) free energies due to mutations, predicting nonspecifically bound ions (BION^12^ method), DelPhiPKa^37^, ^38^ method and webservers for predicting pKa's in proteins, RNAs and DNAs, and calculating the electrostatic forces between macromolecules (DelPhiForce).^39^, ^40^

## Methods

DelPhi C++ is a complete renovation of DelPhi FORTRAN 95 version.^26^ It adopts the same user interfaces (UI), but is easier to maintain, easier to be incorporated in or to integrate with other third‐party software, without jeopardizing its efficiency when solving the Poisson‐Boltzmann Equation (PBE). It can be downloaded from http://compbio.clemson.edu/delphi.

In this section, the architecture of DelPhi C++, together with its new features, will be demonstrated. Interested users and developers are also directed to the online DelPhi developer manual http://compbio.clemson.edu/delphiDir/developer-manual/ for more details.

### Overall description of DelPhi C++ code architecture

DelPhi C++ is an object‐oriented code. The overall code architecture is built over three base classes: (a) an IO class *CIO* for diverse inputs and outputs; (b) a twin abstract classes *IDataContainer* and *IDataMarshal*, which allow variables of various types, including user‐defined variables, to be shared among multiple classes; and (c) an abstract class *IAbstractModule*, which provides a prototype for all task‐related derived classes, each of which carries out a particular task. Details about the code architecture are provided in the supplementary material.

### Parallelization schemes and memory distribution

One of the most important new developments of DelPhi C++ is that the new version, v.8.4, supports three types of implementations (regular single‐CPU, multithreaded, and multi‐CPU parallel). They are integrated in one set of code (details are provided in the supplementary material). DelPhi users can now generate executable of any of these three implementations from the same set of code. All three implementations produce results at the same precision. A guideline of the choice of a particular implementation is provided here: In case of relatively small system requiring grid size of less than 300^3^ grid points, the best choice is the regular single‐CPU implementation. For a medium‐size problem requiring more than 300^3^ up to approximately 600^3^ grids, OpenMP multithreaded parallel implementation is most suitable, and can be run on either a PC or a Linux cluster. When the number of grid points is higher than 600^3^, it is a large‐size problem with a high memory demand, which may exceed the capacity of most PCs. Given its limited computing power, the execution time to solve such a large‐size system on a single PC could be intolerably long. It is advised to solve such types of cases using a high‐performance computing (HPC) cluster, which is usually equipped with thousands or even tens of thousands of CPUs. It is suggested to use the MPI multi‐CPU implementation to utilize computing power and memory of multiple computing nodes on the HPC cluster to significantly accelerate the calculations.

The parallelization schemes carried out in OpenMP multithreaded and MPI multi‐CPU implementations are described in the supplementary material (Supporting Information Fig. S1; Table S1 and S2). An important component is the new memory distribution technique applied in MPI multi‐CPU protocol that reduces the memory requirements in case of very large systems requiring more than 600^3^ grid points (Supporting Information Fig. S2).

### Definitions of testing parameters

The following quantities are used to assess the performance of OpenMP‐ and MPI‐parallelized implementations of DelPhi C++:

*Speedup* is defined to be the ratio of the execution time of a single‐CPU program to the execution time of a (OpenMP‐ or MPI‐) parallelized program running on multiple CPUs. The higher the speedup is, the faster the parallel program is. In principle, a linear speedup is expected in the best case, that is, speedup equals the number of CPUs being utilized. However, in practice, it is known by the Amdahl's Law that, for any well‐designed parallel algorithm, linear speedups can be achieved only when the parallel program runs on small numbers of CPUs, while the speedup eventually reaches a peak value and plateaus out when the parallel program runs on large numbers of CPUs.

*Efficiency* is defined to be the ratio of the speedup to the number of adopted CPUs. It estimates how well‐utilized the CPUs are in solving the problem, compared to how much effort is spent in communication and synchronization. The efficiency varies between 0 and 1. Linear speedup corresponds to the highest efficiency of 1, while an efficiency close to 0 indicates that most efforts of CPUs are consumed by communication and synchronization.

*RAM reduction percentage* (RRP) is defined to be the ratio of the amount of RAM used by one CPU, which utilizes the largest amount of memory among all CPUs in an MPI‐parallelized program, to the amount of RAM used by a single‐CPU program. One goal of the MPI‐parallelized program is to reduce the high‐memory requirement on one computing node when solving the PBE for a large system. In the MPI‐parallelized implementation, instead of requiring large amount of memory on one computing node (typically the master node), the required memory is distributed as evenly as possible on multiple computing nodes. This memory reduction allows the MPI‐parallelized program to be executed not only on the computing nodes equipped with large memory, but also on moderate clusters with less memory. RRP is a quantity used to measure the reduction of memory required to run the MPI‐parallelized program.

## Results

Benchmarks reported in this section were all performed on the HPC cluster *Palmetto* https://www.palmetto.clemson.edu/palmetto/ located at Clemson University. Palmetto cluster has over 2020 computing nodes with various hardware configuration. Some nodes are more powerful than the others in terms of CPUs and RAMs. In order to execute the program in a timely manner, benchmarks were conducted on various types of computing nodes. Detailed hardware configuration of adopted computing nodes will be provided in each subsection.

Single‐CPU and multi‐CPU DelPhi programs were tested on examples of small‐, medium‐, and large‐size cases. All programs have been assured to produce identical electrostatic potentials and energies on all tested examples. Therefore, here we are only focusing on comparing computational costs. In order to provide accurate comparison, each identical run was repeated three times, and the averaged running time is used for the reported speedup and efficiency.

#### *Parallelization (speedup, efficiency, and memory usage benchmarking)*

When the system size is larger than 300^3^ grids, it is advised to use either OpenMP or MPI version of the DelPhi program. Here, we test the efficiency of OpenMP and MPI implementations using a large protein (PDB ID: 4UDF^41^) of more than 38,000 amino acids and 300,000 heavy atoms. Both linear and nonlinear PBEs are solved and the three aforementioned quantities: speedup, efficiency, and memory reduction percentage, are benchmarked. The benchmarking was performed on a typical everyday load of Palmetto supercomputer Linux cluster, each run was repeated three times, and results averaged.

**Figure 1:**
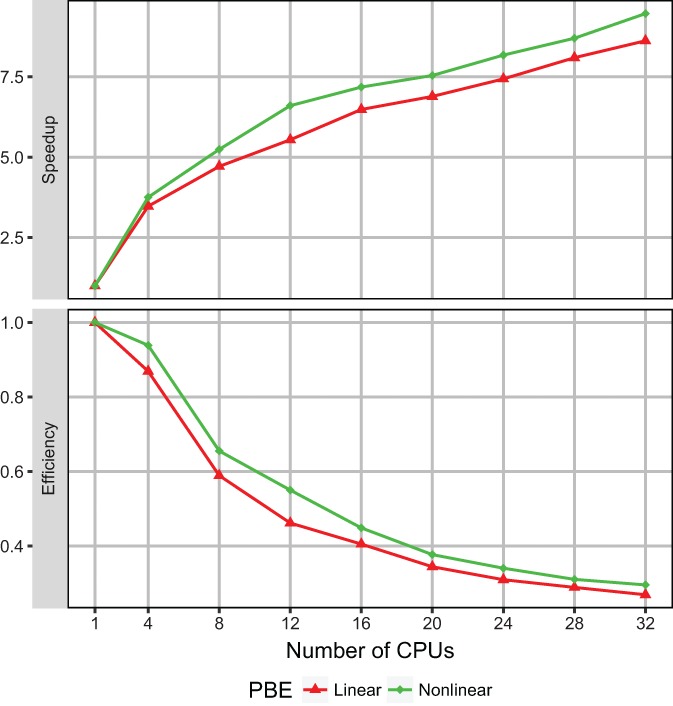
Numerical benchmarks of solving the Linear and Nonlinear PBEs on aprotein with PDB ID: 4UDF using Open MPDelphi. Speedup (top panel) and efficiency (bottom panel). [Color figure can be viewed at wileyonlinelibrary.com]

The OpenMP DelPhi program is tested first. The OpenMP program is a multithreaded version of the single‐CPU program. It utilizes the computing power of multiple CPUs equipped on one computing node and thus uses the same amount of memory as that used by the single‐CPU implementation. Therefore, only speedup and efficiency were benchmarked for the OpenMP DelPhi implementation. The parameter *scale* was fixed to be 1.0 (i.e., one grid per angstrom), resulting in a total of 561^3^ ≈ 177 million grids. The size of the problem is selected to represent a typical medium‐size problem. For benchmarking, we selected a particular computing node, Dell R740 server, Chip model Intel Xero 6148G, which is equipped with 40 cores allowing us to utilize up to 32 CPUs. The obtained speedup and efficiency when solving the linear and nonlinear PBEs on the protein 4UDF are presented in Figure 1.

Figure 1 shows that the OpenMP DelPhi implementation significantly reduces the computational cost of solving the linear and nonlinear PBEs. The achieved speedups on 32 CPUs are 8.6 and 9.4 for linear and nonlinear PBEs, respectively. In terms of absolute execution time, the running time of the single‐CPU implementation is 2 h, while the OpenMP implementation completes the run in 11 min, when solving the linear PBE. Similarly, the execution times for single‐CPU and OpenMP implementations are 3.5 h and 22 min, respectively, for solving the nonlinear PBE.

One should bear in mind that the OpenMP program does not reduce the RAM usage compared with the single‐CPU program since it can only utilize the memory equipped on the same computing node. This makes the OpenMP implementation most suitable for solving medium‐size problems with sizes ranging from 300^3^ to 600^3^ grids. When the problem's size is extremely large (> 600^3^ grids), it requires significant amount of RAM and can last for more than a day. In this case, it is advised to utilize the MPI DelPhi implementation to handle the most computationally expensive tasks.

**Figure 2:**
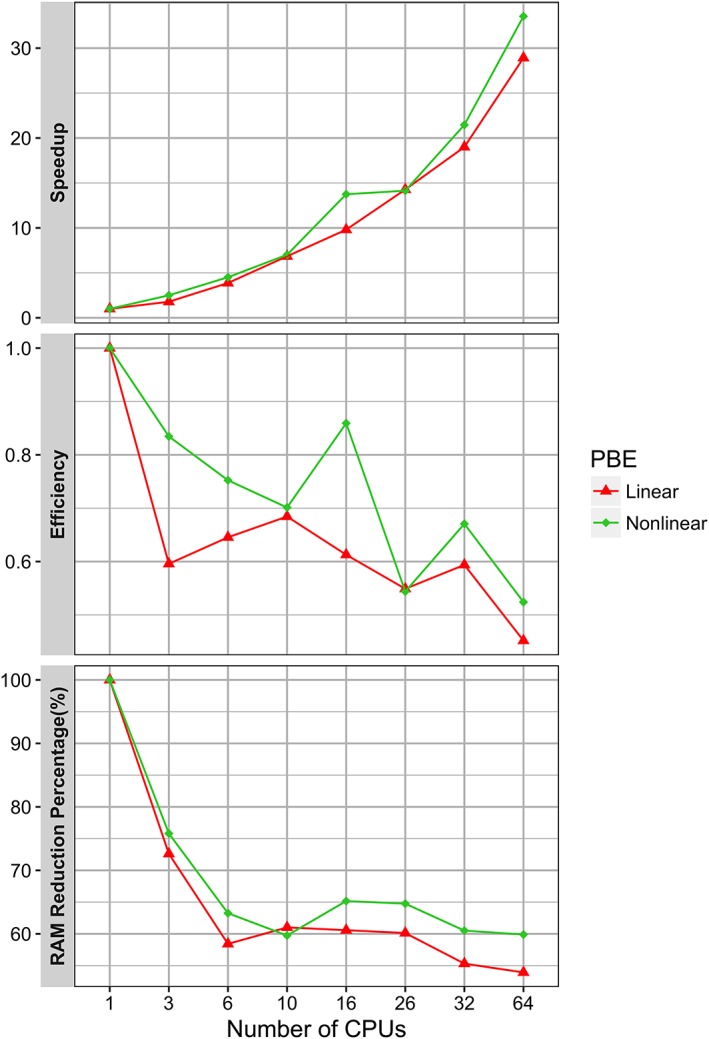
Numerical benchmark of solving the Linear and Nonlinear PBEs on aprotein with PDB ID: 4UDF using MPI Delphi. Speedup (top panel), efficiency(middle panel) and RAM reduction percentage (bottom panel). [Color figure can be viewed at wileyonlinelibrary.com]

In order to demonstrate the performance of the MPI DelPhi implementation, the parameter *scale* is doubled (scale = 2.0) in the example of protein 4UDF, resulting in a total of 1123^3^ ≈ 1.4 billion grids. The MPI DelPhi program is then used for solving the linear and nonlinear PBEs. Here, we benchmark speedup, efficiency and RRP. It was done by allocating one CPU per computing node for all MPI tests. Various combinations of computing nodes on the Palmetto cluster were explored to examine the robustness of results. The achieved benchmarks of speedup, efficiency, and RRP are shown in Figure 2.

Several important observations can be made analyzing the results from MPI benchmarking. First, since the MPI implementation uses CPUs across multiple computing nodes, this allowed us to use more CPUs (as compared with OpenMP benchmark). As a result, the MPI implementation achieves higher speedups (29 and 34 times faster for solving linear and nonlinear PBEs, respectively, on 64 CPUs), compared with those obtained by the OpenMP implementation (8.6 and 9.4 times faster for solving linear and nonlinear PBEs, respectively, on 32 CPUs). Second, the algorithm implemented to parallelize the molecular surface construction works more effectively when the number of utilized CPUs is proportional to a power of two (data not shown). In this case, the algorithm splits the computational domain into subdomains evenly in x‐, y‐, and z‐directions so that the workload on each CPUs is well balanced. Third, the RRP of the MPI implementation does not decrease proportionally as the number of CPUs increases. It decreases rapidly when a small number of CPUs is used and approaches to its minimal value around 55%‑60% as the number of CPUs increases. The peak usage of RAM in the MPI program is found to occur after solving the PBEs for potentials at all grids (data not shown). At this point, the calculated potentials are collected from distributed CPUs and assembled into one piece by one CPU, and then distributed again to be saved onto multiple computing nodes. It is implemented in this particular manner in order to lower the data traffic among CPUs and balance the efficiency and RAM consumption on one computing node.

**Figure 3:**
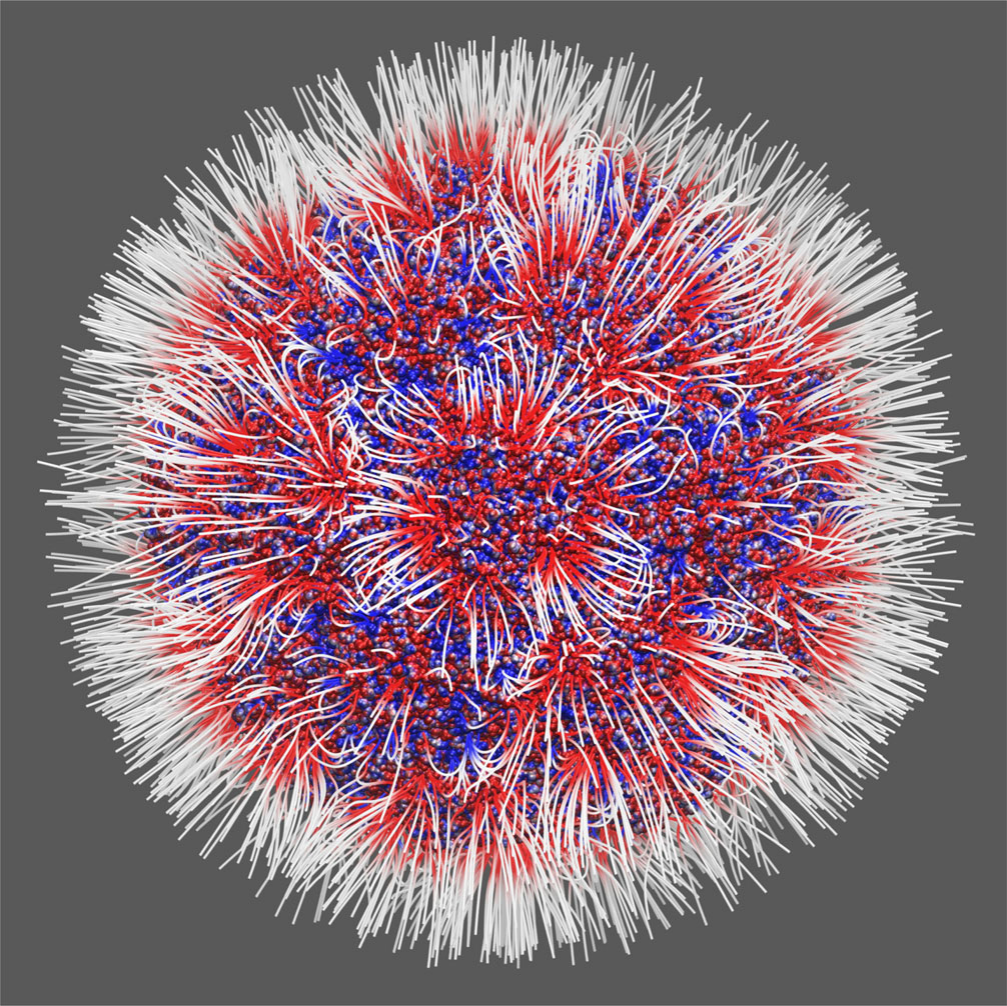
Electrostatic field lines human parechovirus: red and blue correspond to negative and positive polarity, respectively. The modeling was done using PDB ID 5MJV, which contains 180 proteins, including 41,880 residues. The diameter of the whole virus is 298 Å.

The above results showed that Delphi C++ (Open MP and multi‐CPUs implementations) can handle large systems in which modeling requires more than billion grid points, both in terms of time of calculations and memory requirements for the computer performing the job. Given that many researchers nowadays are interested in modeling large macromolecules and their assemblages, this development will definitely help. To illustrate the complexity of electrostatic potential and electrostatic field distributions in such large assemblages, in Figure 3, we show the electrostatic field lines generated using Delphi for the human parechovirus. One can see that the distribution is highly inhomogeneous and cannot be modeled without solving PBE.

#### *Assessing DelPhi C++ accuracy*

Achieving high computational speed and extending DelPhi capabilities to handle large systems is an important development, however, it is equally important to deliver correct solutions. To check the accuracy of DelPhi C++ calculated electrostatic features, such as potential, energy, and electrostatic field, we benchmarked it against analytical solutions. The examples are provided in the supplementary material and are available for download from http://compbio.clemson.edu/delphi. The results indicate that depending on geometry, charge distribution and electrostatic quantity being benchmarked, DelPhi C++ can achieve highly accurate solutions even at a scale of 1 grid/Å (Supporting Information Figs. S3, S4, and S5).

### Newly added features and resources associated with DelPhi

In this section, we describe the newly added features in the DelPhi package. They either represent new treatment of a particular component of the framework of PBE or a new quantity delivered via DelPhi modeling. Details are provided in the supplementary material.

#### *Gaussian‐based smooth dielectric function and its applications*

Traditional PBE approaches model the space of macromolecules as low dielectric constant volume cavity immersed into a high dielectric space of water phase. These approaches do not take into account the inhomogeneity of both the macromolecule and the space between solute‐solvent. Here, we specifically do not use the term “molecular surface” since we argue that there is no sharp border between macromolecule and the water. Instead we would like to consider that there is a smooth transition between macromolecule and water phase. Thus, in the Gaussian‐based approach atom, densities are presented as Gaussian density function.^42^, ^43^ Then using the resulting density function, one delivers the dielectric “constant” as a function of space (details are provided in the corresponding references^42^, ^43^) (Supporting Information Fig. S6). Such approach results in a smooth dielectric function that reflects the following physical expectations: low‐packed macromolecular regions as protein's surface are assigned higher dielectric constant compared with the hydrophobic core; the cavities inside the macromolecule are assigned dielectric constants higher than the rest of macromolecule but smaller than bulk water; and there is a smooth transition of the dielectric constant from macromolecule to water, to reflect that surface waters are not so free to move compared with bulk waters. This approach is particularly useful in modeling protein–protein binding^44^ and pKa's modeling.^37^

#### *Mobile ions treatment via born solvation term in PBE*

The above approach does not draw sharp dielectric border between solute and solvent which raises a question about the treatment of the mobile ions in water phase. In traditional approaches, one treats the mobile ions in the water phase via modeling their density with Boltzmann factor. However, this requires a knowledge of what part of the space is pure water and what part is taken by solute. Arguing that there is no sharp border between the solute and the solvent, we introduced an additional term in the traditional PBE. Instead of using geometrical factors to determine what part of the space the mobile ions can be present in, we penalize the presence of mobile ions via a desolvation penalty term which makes it difficult for the ions to enter macromolecule (Supporting Information Fig. S7). Details are provided in the corresponding paper,^45^ where we demonstrate that this approach correctly predicts the salt‐dependence of protein–protein binding.

#### *Zeta‐potential*

In most of the cases, one is interested to compute the electrostatic potential inside the macromolecule or macromolecular complex. However, equally important is the potential in the space outside the macromolecule, particularly referring to Zeta‐potential. Zeta potential is an important characteristic that is used to assess the propensity of aggregation—if the corresponding particles/macromolecules have high Zeta‐potential, they are expected not to aggregate due to mutual electrostatic repulsion. Motivated by such an importance, we developed a new DelPhi option that allows the users to compute both, the distribution of electrostatic potential at a surface located at a user specified distance away from van der Waals surface and the corresponding Zeta‐potential (Supporting Information Fig. S8). Details are described in the corresponding paper.^46^

#### *Surface‐free approach of computing pKa's of proteins, RNAs, and DNAs*

Computing proton equilibria or predicting pKa's of ionizable group has been explored by many researchers. In terms of continuum approaches, all other approaches use the standard PBE protocol that draws a sharp border between solute and solvent. Taking advantage of the Gaussian‐based approach, we developed a protocol that computes pKa's of ionizable groups of proteins, RNAs, and DNAs without defining molecular surface, so termed surface‐free pKa approach^37^, ^38^ (Supporting Information Fig. S9). It was shown to outperform the traditional approaches in predicting pKa's of both wild‐type proteins and mutant proteins. Recently, this approach was updated to include predictions of pKa's of polar groups and to allow for including salt effects.^47^ In the new releases of DelPhiPKa, we demonstrate that our approach outperforms all existing pKa predictors, including explicit water models, in calculating pKa's of Cys residues.^47^

#### *DelPhiForce*

Electrostatic forces are the most long‐range forces in molecular biology, therefore, their modeling is important for understanding various molecular phenomena, perhaps the most relevant being molecular recognition. DelPhi's FRC module was upgraded to allow for more accurate computation of electrostatic potentials at given points in space.^39^ This was combined with scripts that allow the users to compute electrostatic force between two molecules, or in general, between two user‐specified entities (pairs of residues, receptor‐ligand, etc.) (Supporting Information Fig. S10). It was demonstrated that the electrostatic forces not only guide the partners together, but also adjust their mutual orientation prior physical binding.^39^

#### *Computing folding free energy changes due to mutations (SAAFEC method)*

Solvation energy is an indispensable component of the total free energy of folding; therefore, its accurate computing reassures accurate predictions of the folding free energy.^33^ Of particular interest for personalized medicine is modeling of the effect of nonsynonymous variants (amino acid variants or mutants) on the folding free energy.^48^, ^49^, ^50^, ^51^, ^52^ This interest is motivated by the observation that there is a significant correlation between the propensity of a given mutation to be pathogenic and the magnitude of the folding or binding free energy change.^51^ To address such interest, we developed “Single Amino Acid Folding free Energy Changes (SAAFEC) based on a knowledge‐modified Molecular Mechanics Poisson‐Boltzmann (MM/PBSA)” approach^33^ (Supporting Information Fig. S11), which was benchmarked against experimentally measured folding free energy changes provided in ProTerm database.^53^

#### *Modeling protein–protein binding free energy changes (SAAMBE method)*

Similarly, as outlined above, we developed DelPhi‐based approach of computing binding free energy changes caused by amino acid substitutions.^35^ The method described as “single amino acid mutation based change in binding free energy (SAAMBE)” utilizes 3D structure of the corresponding protein–protein complex and utilizes two approaches: sequence‐ and structure‐based approaches. Thus, the method has two components: a MM/PBSA‐based component and an additional set of statistical terms (Supporting Information Fig. S12). Details are provided in the corresponding manuscript.^35^

#### *Modeling protein‐RNA/DNA binding free energy changes (SAMPDI method)*

Protein‐nucleic acids interactions are essential components of cellular interaction networks and frequently are implicated in human diseases.^36^ This motivated us to develop a method “single amino acid mutation binding free energy change of protein‐DNA interaction (SAMPDI)” method, that computes the change of the protein‐DNA/RNA binding free energy caused by mutations (Supporting Information Fig. S13). Details are provided in the original paper.^36^

#### *Multiscale sampling method (MSSM)*

The multiscale approach is capable of modeling the binding process between large and small biological objects.^54^ The MSSM specifically targets efficiency improvements in the electrostatic energy calculations. We developed a novel algorithm that first calculates electrostatic energy at a course‐grained resolution of the entire system. Then, it transfers the information from the entire environment to a focused local region of interest and calculates the electrostatic energy at a significantly finer resolution. Based on the precalculated energies, the MSSM applies Monte Carlo procedure to evaluate the probabilities of each pregenerated conformation and then evaluates plausible pathway of small object binding onto the large one (Supporting Information Fig. S14).

#### *Delphi webserver*

To further facilitate DelPhi's usage, we developed a DelPhi webserver, using which inexperienced users may submit their jobs without having to familiarize themselves with the DelPhi's stand‐alone program http://compbio.clemson.edu/sapp/delphi_webserver/.^55^ Through self‐navigating system with a help menu, the server provides all necessary parameter files for a DelPhi run. It also allows experienced users to upload their custom parameters to run DelPhi on the webserver. Examples are provided as well (Supporting Information Fig. S15).

#### *DelphiPKa*

It is a webserver that predicts the pKa's of proteins, RNAs, and DNAs.^38^, ^47^ The pKa method was tested against two large databases of experimentally determined pKa's (PPD database: 34 proteins with 302 titratable residues and pKa‐cooperative database: SNase mutants of 109 pKa's). The databases include a variety of cases from strongly coupled titration sites to almost isolated and deeply buried sites. The overall benchmark over three different force field parameters (Amber, Charmm and OPLS) resulted in a RMSD = 0.78pK and RMSD = 1.6pK for PPD and pKa‐cooperative databases, respectively. The preliminary results are among the top in the field. The method is implemented into a webserver, which is easy to navigate and provides various outputs, including predicted pKa's and a structure with proton position assigned at user specified pH. The method and the webserver allow predicting pKa's of proteins, RNAs, and DNAs. Link: http://compbio.clemson.edu/pka_webserver/

#### *SAAMBE webserver*

Single amino acid mutation related change of binding energy (SAAMBE) is a webserver, which addresses the demand for computational tools of predicting the effect of single amino acid substitution on the binding free energy of protein complexes.^34^ It is based on the fast (<< 1 min) and modified MM‐PBSA protocol that is successfully tested and optimized for more than thousand experimental data points from SKEMPI database. Link: http://compbio.clemson.edu/saambe_webserver/

#### *SAAFEC webserver*

Single amino acid folding free energy changes (SAAFEC) is an approach for calculating folding free energy changes caused by missense mutations.^33^ The method is based on weighted MM‐PBSA method with weight coefficients optimized against experimental data from the ProTherm database. Furthermore, the prediction formula is extended to include various biophysical terms to enhance the performance. Link: http://compbio.clemson.edu/SAAFEC/

#### *SAMPDI webserver*

It provides fast and accurate predictions of the effects of single amino acid substitution on the binding free energy of protein‐DNA complex.^36^ This method utilizes modified molecular mechanics Poisson‐Boltzmann Surface Area (MM/PBSA) approach along with an additional set of knowledge‐based terms delivered from investigations of the physicochemical properties of protein‐DNA complexes. An important feature is applying DelPhi's Gaussian‐based smooth dielectric function to calculate the change of solvation energy. Link: http://compbio.clemson.edu/SAMPDI/

#### *DelPhiForce webserver*

This web server implements DelphiForce, which is a DelPhi‐based script, that allows one to calculate the electrostatic force between two objects, such as proteins, DNAs, lipids, small molecules, and so forth.^40^ Users can also generate vector representations of electrostatic force between the two objects. Link: http://compbio.clemson.edu/delphi-force/

## Conclusions

In this work, the most recent developments of the scientific software DelPhi are presented. The new DelPhi program has become a package, which allows users to generate single‐ and multi‐CPU executables from one source code. The newly object‐oriented programming design, as well as the newly added features, upgrade the DelPhi program from a single CPU PBE solver to a multimodel and multisolver platform, preparing it for more complicated computational tasks in the future.

## Supporting information


**Appendix S1**: Supporting Information
