# Supplementary material for: DelPhi Suite: New Developments and Review of Functionalities
Source: J Comput Chem. 2019 Jun 25;40(28):2502–8. doi: 10.1002/jcc.26006 (PMC6771749; doi:10.1002/jcc.26006)
Supplement: Supplementary file 1 — Appendix S1: Supporting Information [file JCC-40-2502-s001.pdf]

Supplementary Material

DelPhi suite: New developments and review of functionalities

Chuan Li, Zhe Jia, Arghya Chakravorty, Swagata Pahari, Yunhui Peng, Sankar Basu, Mahesh Koirala, Shailesh Kumar Panday, Marharyta Petukh, Lin Li, Emil Alexov

Overall description of DelPhi C++ code architecture

DelPhi C++ offers three base classes – an IO class *CIO* for diverse inputs and outputs, a pair of abstract classes *IDataContainer* and *IDataMarshal* for unified data encapsulation and transaction, and an abstract class *IAbstractModule* for flexible model-solver pairing mechanism. These base classes together lay the foundation of DelPhi C++ and allow derived classes to be built upon them for specific tasks. One shall be aware of that no object can be realized to abstract classes, such as *IDataContainer*, *IDataMarshal*, and *IAbstractModule* in OO programming. Instead, derived classes inheriting the abstract classes must be constructed first, and then objects to these derived classes can be realized.

The class *CIO* is the one providing functions to let DelPhi C++ interacts with users. These functions include functions to check input file formats, read a file line by line to obtain useful data, store data to appropriate structures, and write the calculated values into files with desired formats. DelPhi C++ writes files in standard formats, which can be recognized by software, such as VMD[1] and Chimera [2] for 3D structure visualization. One can refer to the online DelPhi C++ user manual http://compbio.clemson.edu/downloadDir/delphi/delphi_manual8.pdf for a list of input/output files (in various formats) supported by DelPhi C++ V8.1. Supports to new files can be either added to the class *CIO* or accomplished by introducing new IO classes inheriting the class *CIO*, in the future.

The next base class is the twin abstract classes *IDataContainer* and *IDataMarshal*, which allow variables of various types, including user-defined variables, to be shared among multiple classes. One can view the class *IDataContainer* a "cargo vehicle" carrying "cargoes" (variables) from one class to another for transaction, while the class *IDataMarshal* is a "shipping list" which determines what cargoes loaded in the vehicle and their "initial quantities" (initial values). Cargoes loaded in the vehicle *IDataContainer* are all stored in a unified data structure *map<string, boost: any>*, where *map* is an associative container template provided by the C++ Standard Library (STL). Its first parameter is an identification key of *string* type, i.e., element's name, and its second parameter is the mapped values storing the content associated to the key. These values are uniformly stored in an object of the type *any* provided by the *boost* library. The vehicle *IDataContainer* also provides accesses (functions) to upload/unload the cargoes so that the quantities of cargoes (variable values) can be changed from one class to another. Given the variety of the variables, *IDataContainer* provides three types, by *value*, by *reference*, or by *pointer*, of the accesses to the variables.

For a given DE model, a pair of derived classes inheriting *IDataContainer* and *IDataMarshal*, respectively, must be constructed to specify associated variables for describing and solving this

model. In DelPhi C++ V8.1, the two derived classes for solving the PBE model are *CDelphiData* and *CDelphiDataMarshal*. *CDelphiDataMarshal* defines and initializes variables used for describing and solving the PBE model, while *CDelphiData* stores these variables and delivers them to other classes. One can follow the same fashion to construct new pairs of derived classes to integrate new models in DelPhi C++.

The last base class is the abstract class *IAbstractModule*, which provides a prototype for all task-related derived classes, each of which carries out a particular task. *IAbstractModule* enforces taking a pointer to an object of a derived class inheriting the abstract class *IDataContainer* as one input parameter, in order to provide a unified data-sharing interface to all derived classes inheriting the *IAbstractModule*. For example, when solving the PBE model in DelPhi C++ V8.1, there are three major tasks - constructing the molecular surface, solving the PBE for electrostatic potentials by the Successive Over Relaxation (SOR) method, and calculating interested energies. These tasks are carried out by three derived classes, *CDelphiSpace*, *CDelphiFastSOR*, and *CDelphiEnergy*, respectively, all inheriting the abstract class *IAbstractModule*. These three derived classes communicate via a unified interface, an object to *CDelphiData*, while details on how to fulfill their own tasks are hidden inside these derived classes. The unified interface also offers great flexibility to model-solver pairing: one model can be paired with multiple solvers, and one solver can be paired with multiple models.

A collection of objects to the class *CIO*, a pair of derived classes inheriting *IDataContainer* and *IDataMarshal*, and a set of derived classes inheriting *IAbstractModule*, yields a particular *application*, i.e., solving the PBE by the SOR method and calculating corresponding energies, in DelPhi C++. Following the same tactics, new *applications* can be added to DelPhi C++ to make it a multi-model multi-solver platform.

Parallelization schemes and memory distribution

Another merit of DelPhi C++ is that it allows three types of implementations (regular single-CPU, multi-threaded and multi-CPU parallel) to be integrated in one set of code to fulfill the task of solving the PBE model by the SOR method and calculate the corresponding energies on various molecules and proteins. DelPhi users can now generate executable of any of these three implementations with no additional effort. All three implementations produce identical calculated results, while depending on the size of a given problem, these three implementations are most suitable for solving PBE on various sizes of molecules and proteins. A guideline of the choice of a particular implementation is provided here. In principle, the computational cost of solving the PBE on a particular molecules and proteins is determined by the number of grid points per direction in the *x*-, *y*-, and *z*- directions. Roughly speaking, when the number of grid points per direction is less than 200, it is considered a relatively small-size problem, which can be effectively solved by the regular single-CPU implementation running on a modern Personal Computer (PC). When the number of grid points per direction is between 300 and 600, it is considered a medium-size problem which requires more memory and costs more time to execute. In this case, the OpenMP multi-threaded parallel implementation is most suitable, and can be run on either a PC or a computing cluster. When the number of grid points in higher than 600, it is a large-size problem with high memory demanding which may exceeds the capacity of most PCs. Given its limited computing

power, the execution time to solve such a large-size problem on a single PC could be unbearably long. It is advised to give this type of problem to a High-Performance Computing (HPC) cluster, which is usually equipped with thousands or even tens of thousands of CPUs to solve. In this case, it is suggested to use the MPI multi-CPU implementation to put together the computing power and memory of multiple computing nodes on the HPC cluster to significantly accelerate the calculations.

The following outlines parallelization schemes carried out in OpenMP multi-threaded and MPI multi-CPU implementations.

Parallelization schemes in OpenMP multi-threaded implementation

OpenMP is an Application Program Interface (API) which provides multi-threaded, shared-memory paradigm for splitting the task(s) of a program into multiple sub-tasks, and each of these sub-tasks can be run on an available CPU in parallel. To this end, OpenMP provides a set of compiler directives and library routines to break down one single time-consuming task, usually a single or multi-layer loop in the code, to multiple sub-loops. All sub-loops share the same chunk of memory initially assigned to the program. Each sub-loop is attached to an individual thread for execution in parallel. At the end when all sub-loops are finished, results obtained by sub-loops are assembled into a final result.

OpenMP is one of the most straightforward methods for parallelizing a single-CPU program. Given the fact that most modern PCs are equipped with processors consisting multiple processing units, single-PC users can appreciate the OpenMP-parallelized program to utilize all available CPUs on one single PC to accelerate the execution of a program heavily loaded with calculations, such as solving the PBE on three-dimensional molecules and proteins.

One switch, *PARALLEL_OMP*, in DelPhi C++ turns on/off the OpenMP implementation for parallelizing the most time-consuming loops. When it is on, additional compiler directives and library routines from OpenMP are compiled together with DelPhi C++ source code automatically, to produce an executable, when running, detecting and utilizing all available CPUs for maximum efficiency.

Parallelization schemes in MPI multi-CPU implementation

MPI is a library of Message Passing Interfaces which provides another paradigm, allowing a single-CPU program to be parallelized and executed across multiple CPUs positioned on one or multiple computing nodes connected via high-speed cables. MPI also provides routines to utilize distributed memory on these computing nodes for one parallelized program. Because of the advantages of MPI offers, MPI is ideal for utilizing larger amount of memory and many CPUs to accelerate the execution of a program to solve problems with extremely high computing demands (time and memory usage) and has been widely adopted by HPC cluster users.

The Fortran version of DelPhi, DelPhi 90/95 has been parallelized by MPI, resulting in the first parallel version of DelPhi program - PDelPhi. One can find it at http://compbio.clemson.edu/delphi. In PDelPhi, the three major tasks, namely, constructing the surface of a given molecule/protein, iteratively solving the PBE equation, and calculating

corresponding energies, are parallelized via various specially-designed computational algorithms for faster calculations. PDelPhi has achieved tremendous success in terms of accelerating the calculations of solving the PBE on large proteins or bio-complexes. It was found that PDelPhi, when running on ~ 100 CPUs, is dozens of times faster than the single-CPU DelPhi 90/95, when solving the PBE on proteins consisting of ~ 500,000 atoms[3-4].

In most recent development of DelPhi, DelPhi C++, new algorithms for parallelizing surface construction and evenly distributing the memory usage are introduced in the MPI-parallelized DelPhi C++ implementation. Moreover, in order to accommodate new data structures used in DelPhi C++, a public virtual function *mpi_run* is added to the abstract class *IAbstractModule*, so that derived task-related classes inheriting from *IAbstractModule* now have a unified individual MPI-implementation entry point.

Like the OpenMP implementation, the MPI implementation can be turned on/off by a switch *PARALLEL_MPI* in DelPhi C++ V8.1. In what follows, these new algorithms, together with how the three major tasks are parallelized, will be presented.

Parallel Space Module

The space module constructs the molecular surface and assigns dielectric constants based on the property of the grid areas. When using single CPU, this process can take more than 30% of the computing time for most proteins. To distribute the computation load over multiple CPUs, we divided the molecule spatially into subspaces with buffer area between them (as shown in Fig. S1), similar to many molecular dynamics software packages [5-7]. Buffer zones that are wider than the radius of the largest possible atom in the local area is added to each subspace to ensure consistency with all neighboring subspaces. All subspaces have the same size, and the size is optimized for efficiency based on the size of the molecule and number of worker nodes. During the calculation, each worker node typically handles multiple subspaces for better load balance. After the calculation, the result of each subspace is resembled to global space. All subspaces have the same size, and the size is optimized for efficiency based on the size of the molecule and number of worker nodes.

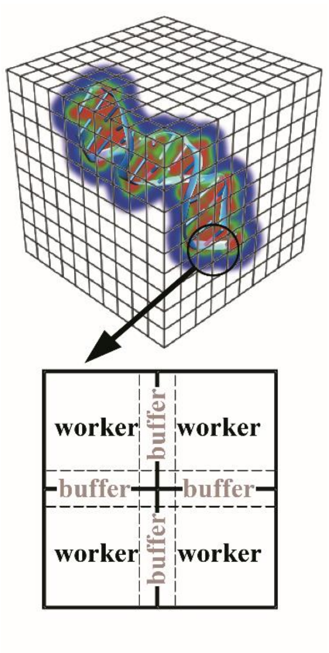

Fig. S1. Parallel approach of space module illustrated with the DNA chain in 9ANT(PDB) from protein data bank.

MPI-Parallelized SOR Solver

The odd-even iteration of the SOR iterative method in the solver class *CDelphiFastSOR* is parallelized using the same algorithm introduced in Refs. [3-4]. Moreover; a couple of changes have been made to accommodate the new OO-design of DelPhi C++.

First, in order to keep the class *CDelphiFastSOR* self-contained, all MPI-related changes, including additional variables for synchronizing intermediate values among CPUs, and new functions carrying out individual MPI calculations on each CPU, are made inside the class

CDelphiFastSOR itself. Not all member functions of the class *CDelphiFastSOR* require to be parallelized. A list of member functions, together with their parallelized cousins, is demonstrated in Table S1. The MPI-related variables and functions are compiled only when the switch *PARALLEL_MPI* is turned on.

Second, one-dimensional arrays for odd-even iteration in DelPhi 90/95 and PDelPhi are substituted with objects to a sequential container *vector* provided by C++ STL in *CDelphiFastSOR*. *Vectors* dynamically allocate contiguous storage for their elements and provide various accesses to the underlying elements. However, the use of *vectors* arises an issue in the MPI implementation, given the awareness that the latest MPI standard has removed C++ bindings so that objects to classes used in the OO programming cannot be delivered from one CPU to another using MPI routines directly. In *CDelphiFastSOR*, a work around of this issue is utilized by associating a pointer to the initial address of the memory where the underlying elements of a *vector*-type object are stored. When the elements are required to be delivered on the sender side, this pointer, instead of the whole object, is given to MPI C binding routines to deliver data, while on the receiver side, this pointer is used to pass the received data to another *vector*-type object.

Property	Single-CPU (& MPI-) Functions	Description
private	*setDielecBndySaltMap* *(mpi_setDielecBndySaltMap)*	Set dielectric and boundary values on grids.
	setCrg *(mpi_setCrg)*	Distribute the charges on atoms obtained from the inputs to all grids.
	initOddEvenItr *(mpi_initOddEvenItr)*	Initialize the odd-even iterations for solving the PBE using the SOR method.
	itrEvenPoints *(mpi_itrEvenPoints)*	Iterates over the odd grids.
	itrOddPoints *(mpi_itrOddPoints)*	Iterates over the even grids.
public	*itit* *(mpi_itit)*	Entry to solve the linear PBE iteratively.
	nitit *(mpi_nitit)*	Entry to solve the nonlinear PBE iteratively.
	run *(mpi_run)*	Main entry of the class *CDelphiFastSOR.*

Table S1. Member functions which have single-CPU and MPI- implementations in class *CDelphiFastSOR*.

MPI-Parallelized Energy Calculations

The energy calculation class *CDelphiEnergy* is parallelized in the same fashion how the solver class *CDelphiFastSOR* is parallelized: all MPI-required variables and functions are incorporated

in the class without breaking the encapsulation of the class. A list of member functions and their MPI cousins is presented in Table S2.

Energy calculations are parallelized in *DelPhi C++* via the same tactics implemented in PDelPhi: a loop over a long list of elements is broken down to multiple exclusive sub-loops so that these sub-loops can be carried out on multiple CPUs simultaneously. Results obtained by sub-loops are assembled into one after all sub-loops are complete. There is only one notable difference between parallel energy calculations in *CDelphiEnergy* and that in PDelPhi: the parallelized Coulombic energy calculation is performed in *CDelphiEnergy*, after the potential map is obtained in *CDelphiFastSOR*, while parallelized Coulombic energy calculation in PDelPhi is performed immediately after the surface construction, even before the potential map is calculated. It indeed slows down the overall execution. However, it is worth to make such sacrifice on efficiency in order to keep the encapsulation of the class *CDelphiEnergy*.

**Property**	**Single-CPU (& MPI-) Functions**	**Description**
private	*energy_clb (mpi_energy_clb)*	Entry to calculate coulombic energy in linear cases (the number of media = 1).
	energy_clbmedia (mpi_energy_clbmedia)	Entry to calculate coulombic energy in multi-media cases.
	energy_clbnonl (mpi_energy_clbnonl)	Entry to calculate coulombic energy in nonlinear cases
	energy_clbtotal (mpi_energy_clbtotal)	Entry to calculate total coulombic energy
	energy_react (mpi_energy_react)	Entry to calculation reaction field energy
public	*run (mpi_run)*	Main entry of the class *CDelphiEnergy*

Table S2: Member functions which have single-CPU and MPI- implementations in class *CDelphiEnergy*.

Memory Distribution

To facilitate the calculation of larger molecular systems, we developed a memory distribution system that allows the computing nodes to access data efficiently in varies scenarios. It has two general components, a centralized storage (Fig. S2a), and a distributed storage (Fig. S2b), and both are accessible by all computing nodes. The centralized storage is only directly linked with the master node. Worker nodes need to visit the master mode to access the centralized storage. It has better performance in storing infrequently used parameters and variables. The distributed storage is dedicated for the larger multi-dimensional arrays. It is evenly distributed among all worker nodes. Worker nodes can access the distributed memory after visiting the owner of the partial memory instead of the master node. Each part of the PBE solver requires distinct memory distribution schema to achieve the best performance. Thus, the distributed storage is independent from the underlying computing algorithm, and it allows the data to be re-distributed and transferred for the sake of efficiency. For example, the space construction performs better under spatially divided memory distribution, and the Gauss-Seidel PBE solver works better in the linearly distributed memory access. To allow them to access the memory efficiently, we conduct memory re-distribution while necessary. It transfers the data in dynamic sizes from 1KB to 1024KB depends on the network condition. This adds data transfer overhead, but significantly reduce the computing time by optimizing the memory distribution, especially when the computation costs significant amount of time.

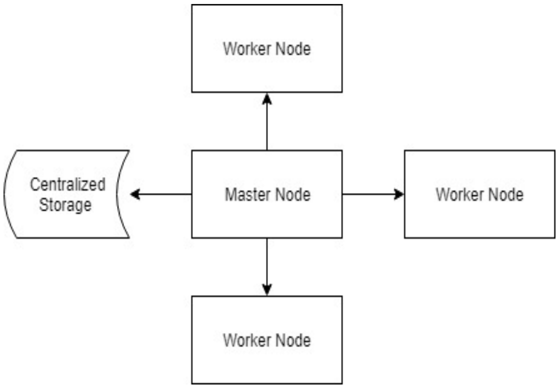

Fig. S2a. Schematic description of centralized storage.

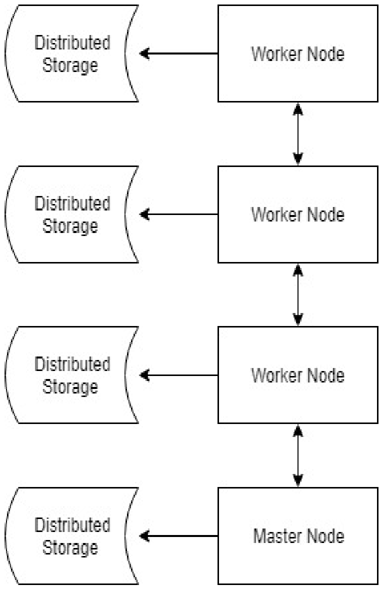

Fig. S2b. Schematic description of distributed storage.

Benchmarking against analytical solutions

In this section we present benchmarking results of DelPhi C++ delivered electrostatic energies and compare them with analytical solutions. This benchmarking is an ultimate test of the accuracy of DelPhi protocol and computer code. Several cases for which analytical solution can be delivered are shown in Fig. S3,4,5. These include electrostatic component of solvation energy of a sphere immersed in solvent (Fig. S3), the total electrostatic energy of two charged in a dielectric cavity (Fig. S4), electrostatic energy of a spherical charge moving across semi-infinite dielectric solvent (Fig. S5A,B) and the same for a spherical charge approaching a cylinder (Fig. S5C,D). Note that semi-infinite dielectric solvent was modeled as a box with dimensions

25x25x25A, and thus represents an approximation. The results clearly indicate that DelPhi C++ delivered energies are matching analytical solutions.

It should be clarified that in case of Fig. S3, electrostatic solvation energy of a sphere, the datapoint at scale=0.5 [grid/A] in case of sphere radius equal to 1A is not provided in the graph. The reason is that finite-difference algorithm requires the size of the object to be larger than the grid resolution (scale = 0.5 [grid/A] corresponds to grid resolution of 2A). Details about Fig. S4 are provided in the original paper [8].

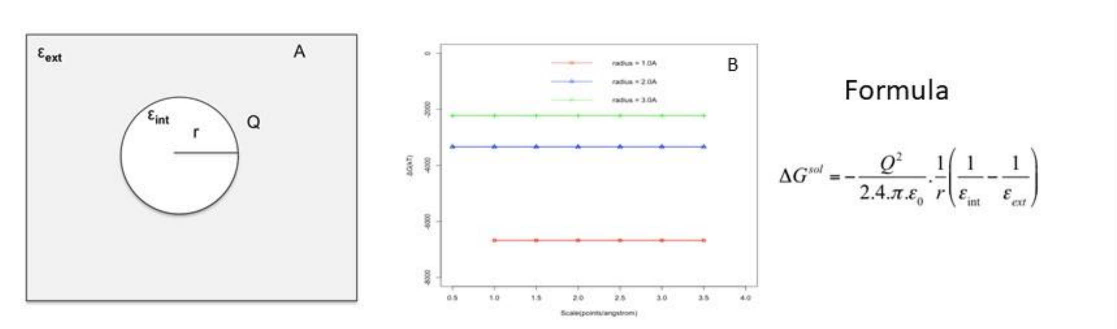

Figure S3(A) Schematic illustration a charged sphere with low dielectric constant is inside a media with a high dielectric constant.(B) Electrostatic component of solvation energies calculated by Delphi against the analytical solution (solid lines) at different sphere radius “r”. Q=10[e], ε_{in}=4, ε_{ext}=80.

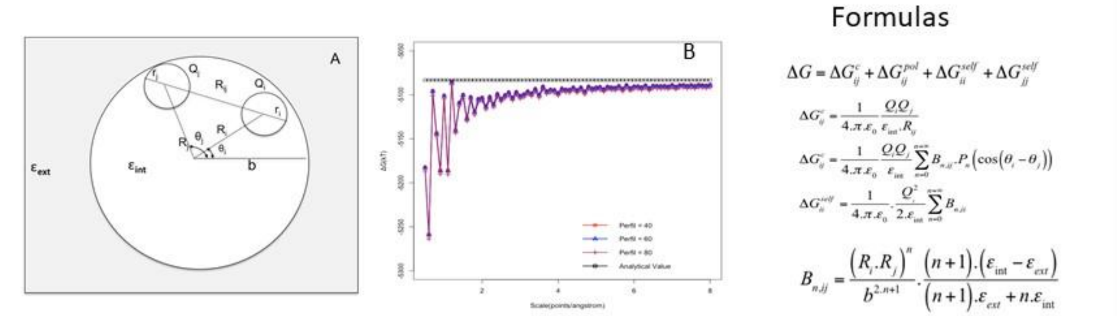

Figure S4(A) Schematic illustration of a cavity with low dielectric constant is inside a media with high dielectric constant. Two charged atoms are located inside the cavity.**(B)** Electrostatic component of solvation energy obtained from Delphi compared with analytical solution (solid line). Details are provided in ref. 8.

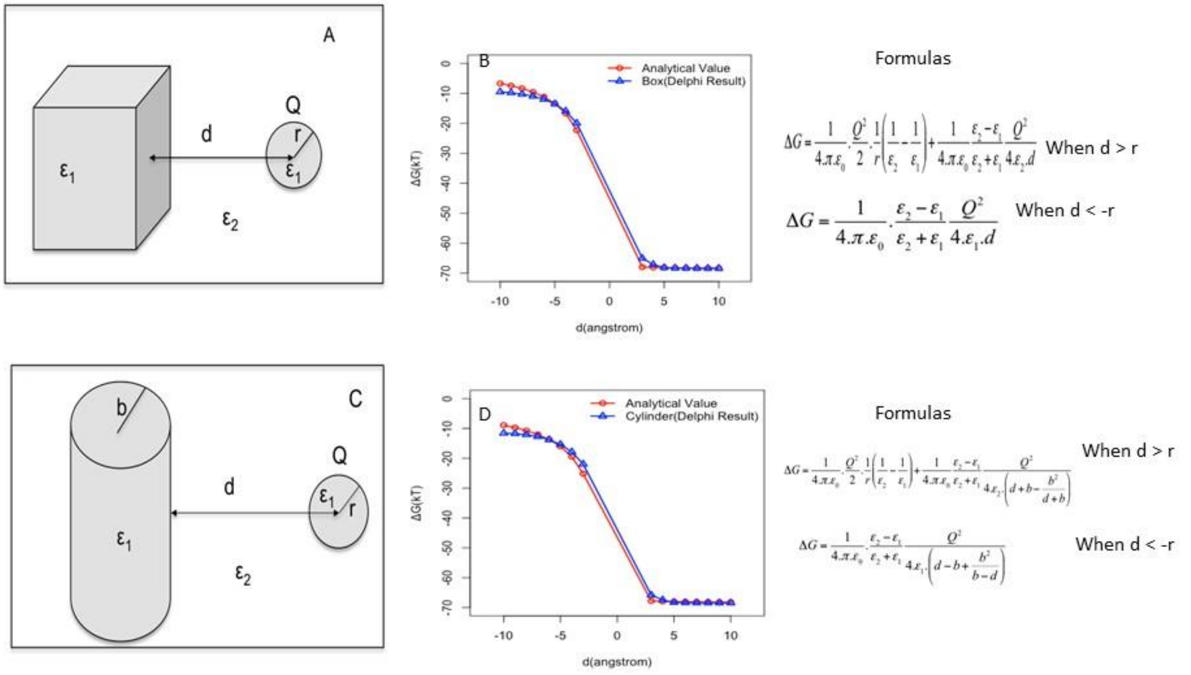

Figure S5**(A) Schematic illustration of a sphere in semi-infinite dielectric plane(Box).** A sphere with low dielectric constant ε_1 is initially positioned in region with high dielectric constant ε_2 and moves into the region with low dielectric constant ε_1 inside the box.**(B)** Electrostatic component of solvation energy derived from Delphi compared with analytical solution.**(C) Schematic illustration of cylinder in two dielectric medium:** A charged sphere with low dielectric constant ε_1 initially positioned with high dielectric constant ε_2 and moves into region of low dielectric constant ε_1 inside the cylinder.**(D)** Electrostatic component of solvation energy obtained from Delphi compared with analytical solution. Here Q=1[e], ε_1=2 and ε_2=80, r=2A, b=10A, length of cylinder=25A.

Newly added features and resources

Below we describe newly added features of DelPhi and newly developed methods and software utilizing DelPhi.

Gaussian-based smooth dielectric function and its applications

One of the most important development of DelPhi in terms of better representation of physical reality within computational model is the introduction of Gaussian-based smooth dielectric function. It allows the solvent-solute to be treated on the same footage. The entire computational space is described via smooth dielectric function (Fig. S6). It was demonstrated that such an approach results in better outcome when computational results are compared with experimental data[9-13].

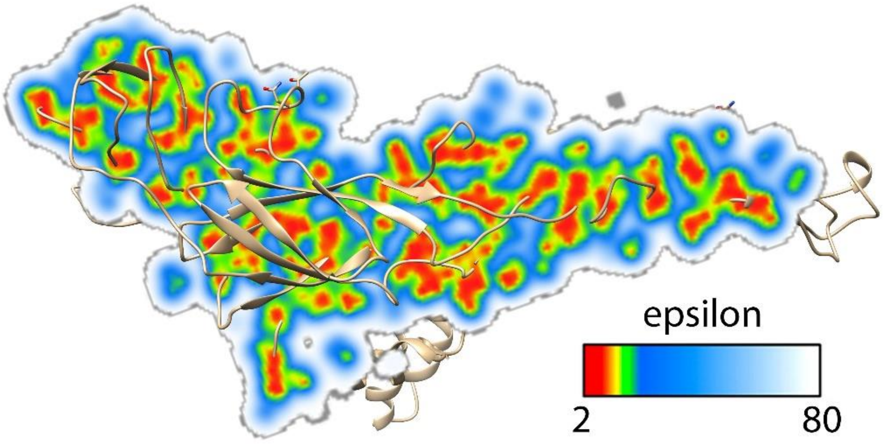

Fig. S6. Dielectric distribution of Envelope protein of Dengue virus (PDB ID: 3J27). The dielectric distribution is calculated by Delphi using Gaussian smooth dielectric function. The color map shows one slice of the 3D dielectric distribution.

Mobile ions treatment via Born solvation term in PBE

When Gaussian-based smooth dielectric function is applied[12], the distribution of ions in solvent is affected by the gradually changing dielectric[11]. Areas close to the bio-molecules typically have lower dielectric constants than bulk solvent. The movement of ions from bulk solvent to these areas is penalized by the dielectric gradient according to the Born equation. The energy penalty from Born equation is converted to distribution probability with the Poisson Boltzmann equation. To prevent the ions entering the bio-molecules, a hard cut-off is applied near the van der Waals surface of the bio-molecules. As a result, the probability of observing a salt ion gradually reduces from the bulk solvent to the areas near the bio-molecules, as shown in Fig. S7.

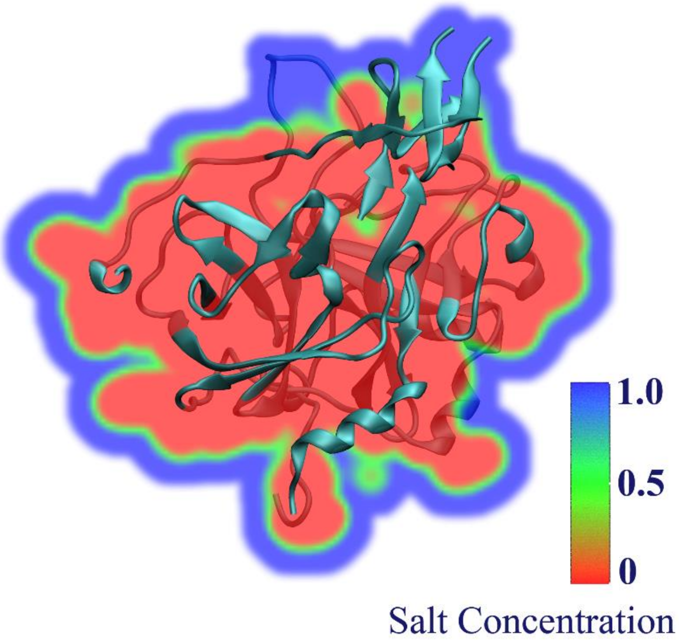

Fig. S7. Relative solvent salt ion concentration of the Hirudin-Thrombin complex (PDB ID: 4HTC). The ion concentration is calculated by Delphi using continuous salt ion concentration method. Ion concentration of 1 means the concentration of bulk solvent, 0 means the absence of ions.

SURFPOT: Computing Zeta potentials

A new feature, called SURFPOT, for computing potential on surfaces outside of the molecule's van der Waals surface was introduced in Delphi [14]. The primary focus of the development was the prediction of zeta (ζ) potential of solutes with complex shapes and inhomogeneous charge distributions in the framework of the Poisson-Boltzmann model. The motivation lies in the fact that experimental measurements of the ζ-potential are rather indirect, in that the measurement of the electrophoretic mobility (μ) is made first and then using simple approximations from the classical electrophoretic theories, applicable to simpler geometries and charge distributions, the ζ-potential is reported. The use of a certain approximation model such as the Helmholtz-Smoluchowski's model, Debye-Huckel model or Henry's model, depends on the concentration of the electrolytes. The electrolyte concentration, as such, influences the formation of an electrical double layer on the surface of the solute on account of its surface charge. But with complexly shaped solutes, the double layer may acquire complex geometries and eventually the applicability of these classical models can get limited.

Our work demonstrated the use of SURFPOT to predict the ζ-potential of 4 proteins and these were compared against their experimental values reported using either of these electrophoretic models. As a function of the pH and the ionic concentration associated with the experimental measurements, we used Delphi and DelphiPka[13, 15] to model the appropriate protonation state of these proteins and computed the surface potential at distances 4-10Å from their van der Waals surfaces. To emphasize that modeling the ionic double layer can have a significantly improve the predictions, we used another Delphi-based package called BION [16-17] to add explicit surface-bound ions to the protein structures. The improvement leads to precise predictions of the ζ-potential and the distance of the surface of the electrical double layer (also called the shear surface) at approximately the Bjerrum length distance from the van der Waals surface (~7-8Å). In entirety, our study showed that by appropriately modeling the protonation state and non-specific surface bounds explicit ions, one can use Delphi's SURFPOT module to predict a protein's ζ-potential (Fig. S8).

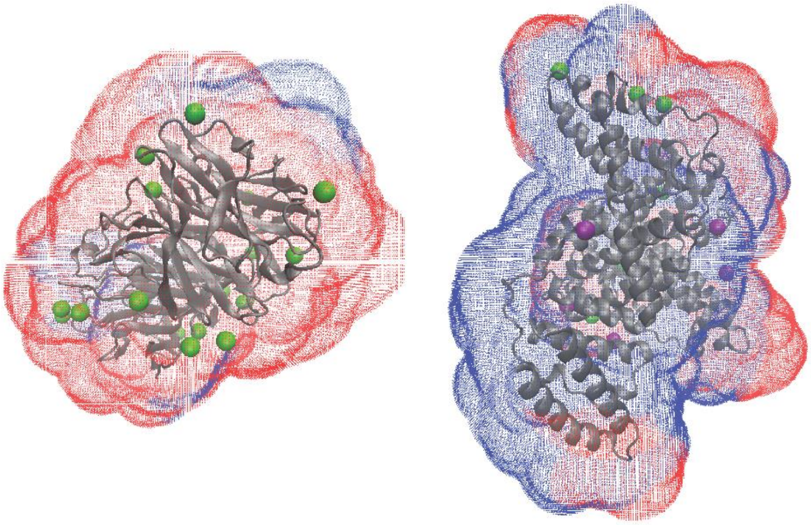

Fig. S8. A visual depiction of the output obtained using the SURFPOT module of Delphi. It shows points that are located tentatively on a shear surface, i.e. a surface at distance 7Å from the van der Waals surface of (left) Invertase from Baker's yeast (PDB ID: 4EQV) and (right) Bovine serum Albumin (PDB ID: 4F5S). A point on a surface is colored according to the electrostatic potential on it; 'Red', 'White' and 'Blue' colors are used to denote a point with a negative, zero or positive potential respectively. The proteins are shown in a cartoon representation and the explicit ions surrounding them are shown as colored beads ('Na' in 'green' and 'Cl' in 'purple). The figures were generated in VMD using a TCL script (available at http://compbio.clemson.edu/downloadDir/ see Surface.tcl) that draws and colors the surface points based on their electrostatic potential as output by the SURFPOT module.

Surface-free approach of computing pKa's of proteins, RNAs and DNAs.

DelPhiPKa can accurately (with a RMSD of 0.77) predict pKa's of ionizable residues in proteins, RNAs and DNAs[18 19]. The use of smooth Gaussian based dielectric function, without the need of determining the solute-solvent boundary, made DelPhiPKa a unique method for calculating pKa's. A new feature of DelPhiPKa is introduced recently, which considers the presence of salt in the

modeling protocol. The salt mobile ions present in the solvent are penalized to enter to the solute phase via a desolvation penalty term which is incorporated in the Boltzmann factor in the PBE framework. Therefore, the presence of salt near protein is determined by a balance between electrostatic interaction and desolvation penalty. A significant improvement has been noticed in the correlation between experimental and DelPhiPKa computed pKa's (having RMSD of 0.74) on taking the salt concentration into account[20]. Another new feature of DelPhiPKa enable the calculation of pKa's of polar residues such as cysteine, serine, threonine and tyrosine. Our recent study demonstrated that DelPhiPKa outperforms all the other existing methods, even the explicit water models, in determining the pKa of cysteine present in the catalytic sites[20]. In Figure S9, three examples are shown where DelPhiPKa accurately predicts the pKa of some ionizable groups, which are strongly influenced by the surrounding electrostatic environment provided by the neighboring groups. For example, in Figure S6a, a salt bridge is formed between Asp70 and His31 because of which there is significant decrease in the pKa value (2.7) of Asp70. In Figure S6b, because of strong interaction with cationic histidine, the pKa of Cys25 (5.5) is lowered. In Figure S6c, in case of triclinic lysozyme, there are 5 backbone NH groups of 4 different residues (Asn27, Trp28, Gly26, Leu25 and Ser24), placed within 4 Å to Ser24. This large positive environment near Ser24 makes the unprotonated form of Ser24 more stabilized and therefore, lower its pKa (9.14).

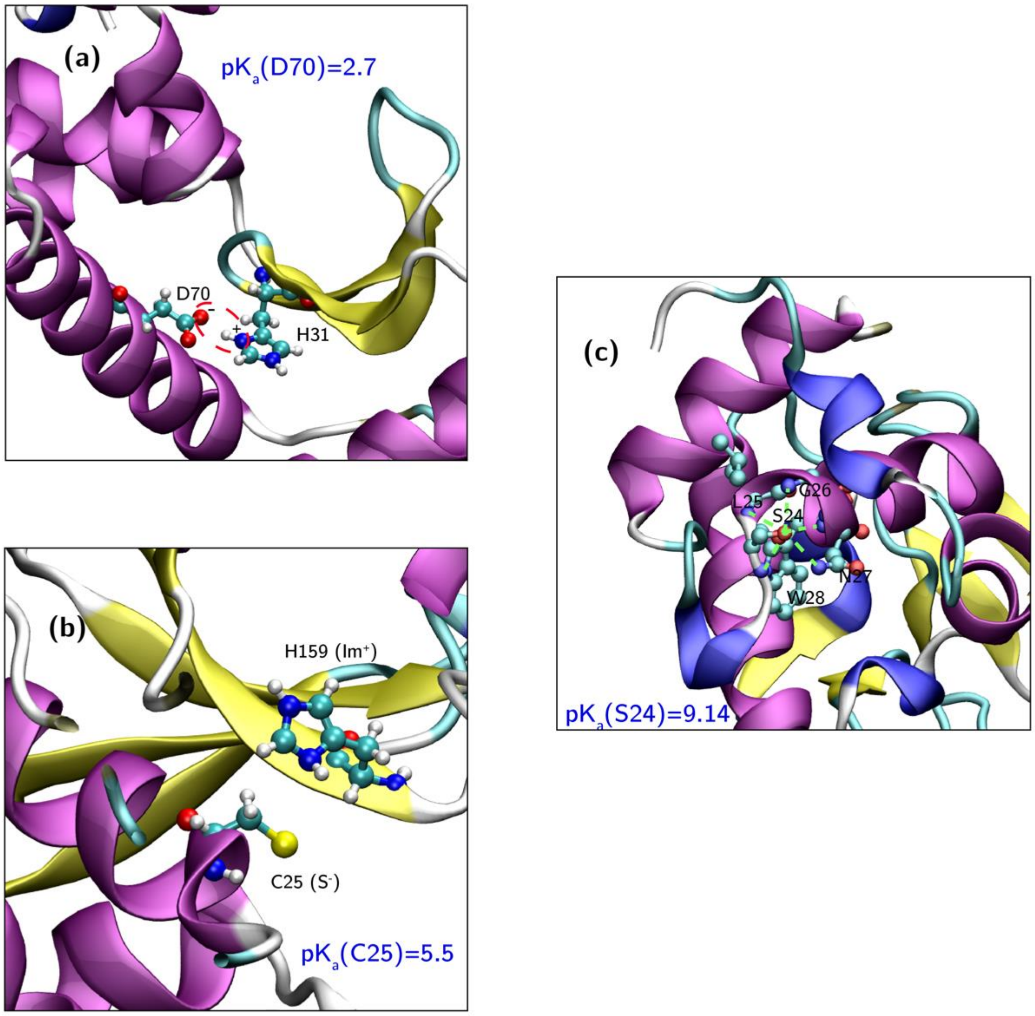

Figure S9: Snapshot of surrounding electrostatic environment near a) Asp70 in T4 lysozyme (PDB ID: 2LZM); b) Cys25 in papain (PDB ID: 1PPN); and c) Ser24 in triclinic lysozyme (PDB ID: 2LZT)

DelPhiForce

DelPhiForce[21] has been developed in the DelPhi package which provide the tool for calculating and visualizing electrostatic forces in biomolecular systems (Fig. S10). It is designed for analysis of protein-protein binding problems. Based on the electrostatic potential calculations from DelPhi, a tri-cubic interpolation method has been implemented in DelPhiForce which derives the electrostatic field and forces. DelPhiForce has been tested against analytical solutions and it has been demonstrated that the corresponding errors are negligibly small at resolution 4 grids/Å. The output of DelPhiForce is in a *.tcl format that support VMD. DelPhiForce web server is available for download from the DelPhi webpage at http://compbio.clemson.edu/delphi-force/[22].

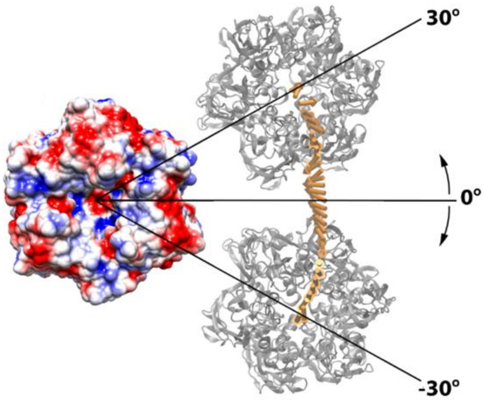

Fig S10. Using DelPhiForce on capsomeres of Paramecium bursaria chlorella virus (PBCV-1) to study the interactions between two capsomeres. One capsomere is fixed at the left and the electrostatic is calculated by DelPhi. The other capsomere (shown in gray) is rotated from -30 to +30 degrees by step of 2 degrees. At each rotated position, the DelPhiForce is used to calculate the binding forces which are shown as orange arrows.

Computing folding free energy changes due to mutations (SAAFEC method)

The folding free energy is directly related to the stability of protein. Mutation can affect the stability of the protein and hence, causes various diseases. Therefore, it is important to understand how any given mutation alters the folding free energy. SAAFEC can accurately predict the folding free energy change due to single point mutation [23]. The method is based on a combination of Molecular Mechanics Poisson-Boltzmann (MM/PBSA) approach and a set of knowledge-based terms, provided by statistical study of biophysical properties of proteins. The method also determines the structural changes due to mutation and provides corresponding energy terms and energy minimized structures for both wild-type and mutant proteins. A correlation coefficient of 0.65 has been achieved through benchmarking against 983 experimental data points using

SAAFEC [23]. SAAFEC method can also be used to identify the disease-causing mutations, therefore can be applied to human health related issues. Figure S11 represents the thermodynamic cycle, used to calculate folding free energy change due to mutation for any protein. The difference $\Delta\Delta G=\Delta G_3-\Delta G_2$ is the folding free energy change due to single point mutation.

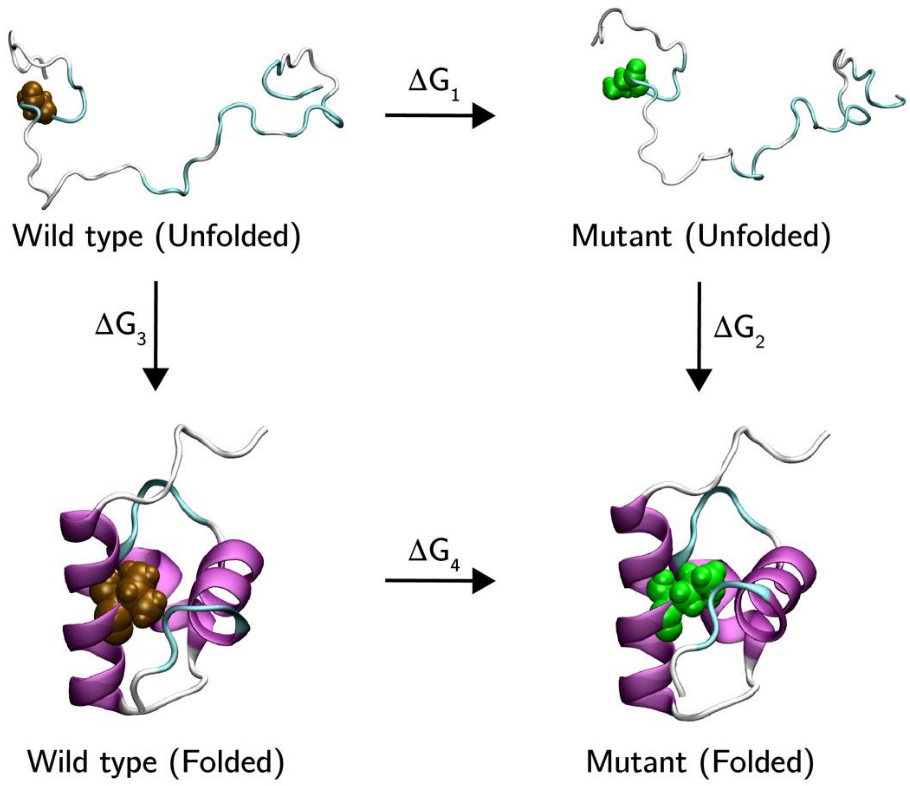

Figure S11: Calculation of folding free energy change due to single point mutation in protein

Modeling protein-protein binding free energy changes (SAAMBE method)

Protein-protein interactions play essential role in all living organisms. At molecular level recognition is affected by multiple factors including macromolecules shapes, charge distribution, conformational flexibility, physico-chemical properties of the interfaces etc. The change in protein sequence such as insertion, deletion or amino acid substitution effect protein binding that potentially can cause a disease. A new methodology termed **S**ingle **A**mino **A**cid **M**utation based change in **B**inding free **E**nergy (SAAMBE) predicts the changes of the binding free energy caused by single amino acid substitution. The method utilizes 3D structures of the corresponding protein-protein complexes and takes advantage of both sequence- and structure-based approaches. The method has two components: a MM/PBSA-based component and an additional set of statistical terms delivered from statistical investigation of physico-chemical properties of protein complexes (Fig.S12). While the method is based on rigid body approach and does not explicitly consider plausible conformational changes caused by binding, the effect of conformational changes on electrostatics is mimicked with amino acid specific dielectric constants. The benchmarking against correspondent experimentally delivered values resulted in a very good agreement

(correlation coefficient 0.624). At the same time the algorithm is fast enough to allow for large-scale calculations (the average time is less than a minute per mutation) and was proved to provides reasonable balance between computational time and details of the modeling

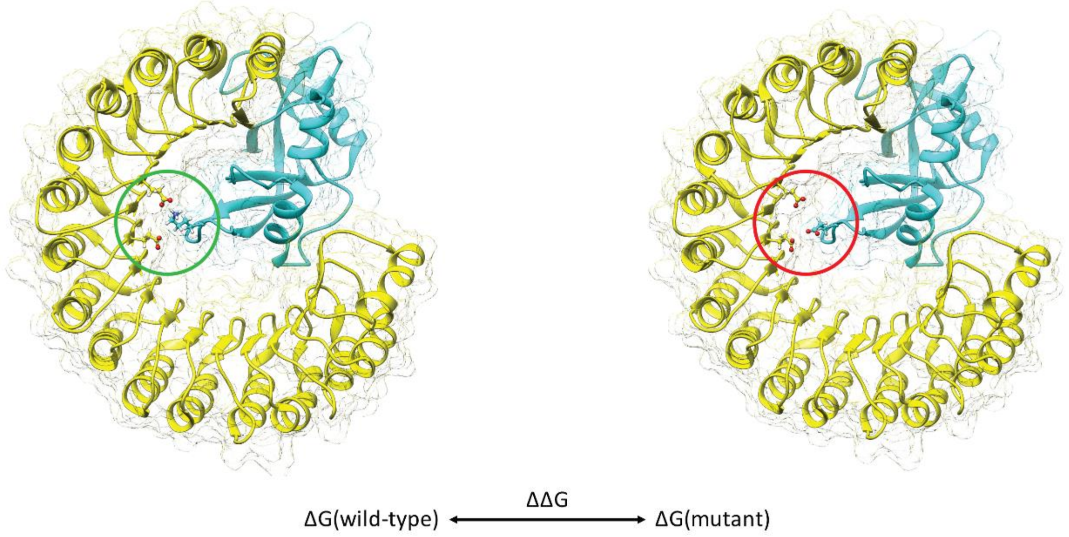

Figure S12. Cartoon presentation of protein-protein complex, where wild type residues are shown within green circle, and mutant residue – in red one.

Modeling protein-RNA/DNA binding free energy changes (SAMPDI method)

Protein-DNA interactions are abundant in the living cells and play crucial roles in different levels of biological processes[24]. Understanding the effects of single mutations in protein-DNA interactions can help us to investigate the human diseases associated with the alteration of protein-DNA binding[25-26]. SAMPDI (**S**ingle **A**mino acid **M**utation binding free energy change in **P**rotein–**D**NA **I**nteraction) approach is capable of performing fast and accurate predictions of binding free energy changes upon single mutations in protein-DNA interactions[27] (Fig. S13). This approach combined modified MM/PBSA approach with set of knowledge-based terms delivered from the physicochemical properties of protein–DNA complexes to achieve accurate prediction results comparing with experimental data[27].

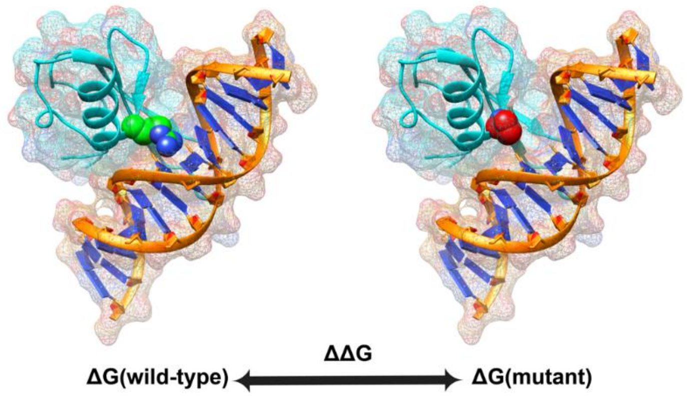

Fig. S13. Calculation of the binding free energy change upon single mutation in protein-DNA interaction.

Multi-Scale Simulation Method (MSSM)

Multi-Scale Simulation Method (MSSM) has been developed to simulate the process of a protein binding to a large system, such as molecular motor protein binding to a microtubule[28], capsomere protein binding to a virus capsid, etc. Utilizing the pre-sampling method and focusing method, the MSSM is able to run fast sampling and energy calculations on large objects, and then perform rigid body Monte Carlo simulations on them. Based on the binding energy calculations including Coulombic energy, polar solvation energy, non-polar solvation energy and Van der Waals energy, the MSSM is designed to simulate the binding pathways, and also to analyze the binding energy funnels[29-30] (Fig S14).

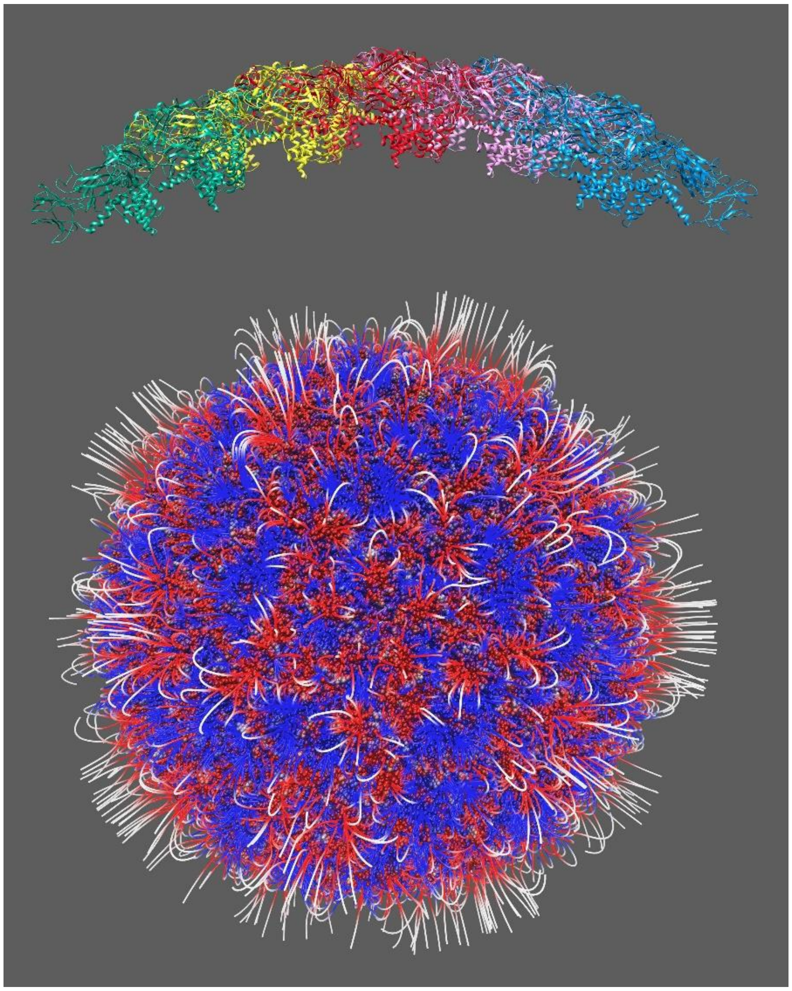

Fig. S14. Schematic of Multi-Scale Simulation Package (MSSP) for Dengue virus. The capsid of Dengue virus (PDB ID: 3J27) is composed by 60 subunits. Each subunit contains three envelope-protein-Es and three small envelope-protein-Ms. One subunit is taken away from the whole capsid and MSSP is utilized to simulate the single subunit binding to the rest of the capsid. The electric

field lines are calculated by DelPhi. One subunit of Dengue virus is shown as shifted and rotated on above the capsid.

DelPhi examples

Delphi examples include several new advanced features as well as several basic ones. Here is a pictorial demonstration of some of them. It starts from the simple case of calculating the corrected reaction field energy of a single charged atom which has also extended to computing the surface electrostatic potential on its van der Waals surface (Fig. S15, top left panel). It is demonstrated that DelPhi calculated solvation energy matches analytical solution up to the second digit after the decimal point at any grid resolutions. Several more examples for cases with analytical solution are provided and it is shown that DelPhi delivered energies are consistently accurate. Furthermore; DelPhi energy calculations are demonstrated for the case of a protein molecule along with providing an interface to create and visualize its electrostatic surface in Chimera and Pymol using the 'cube' (an ascii text) format to output the surface potential values (Fig. S15, top row, middle panel). Electrostatic focusing (Top right panel) is another crucial addition to the examples wherein, by opting the 'phimap' option, one can calculate electrostatic potential and electric field for a particular region with high precision with a reduced computational time using the 'phimap' option. The treatment of salt within the premise of the Gaussian smooth dielectric functionality (Fig. S15, middle row, left panel) is yet another important feature to portray a more realistic picture of the biomolecular system. One can also compute the binding energy (and other associated energy terms) in the context of a biomolecular interaction, say, protein-ligand (Fig. S15, middle row, middle panel). Delphi can also be extended to generate spherical dielectric cavities with charges placed inside (Fig. S15, middle row, right panel). This can be useful in the context of probing nano-protein interactions. Yet another advanced functionality to analyze peptide-membrane interaction has also been incorporated (Fig. S15, bottom right panel). A recent feature to compute electrostatic potential on user specified surface is also illustrated (Fig. S15, bottom middle). Lastly, an example demonstrating the electrostatic complementarity (EC) between surface potential at a protein-protein interface has also been incorporated which has been proven to be one of the major parameters to probe both binding and folding. Figures were created by RasMol, PyMol, Chimera and the bioinformatic toolbox of MATLAB. All examples are vividly explained in the distribution with README files, and user-friendly command-line options.

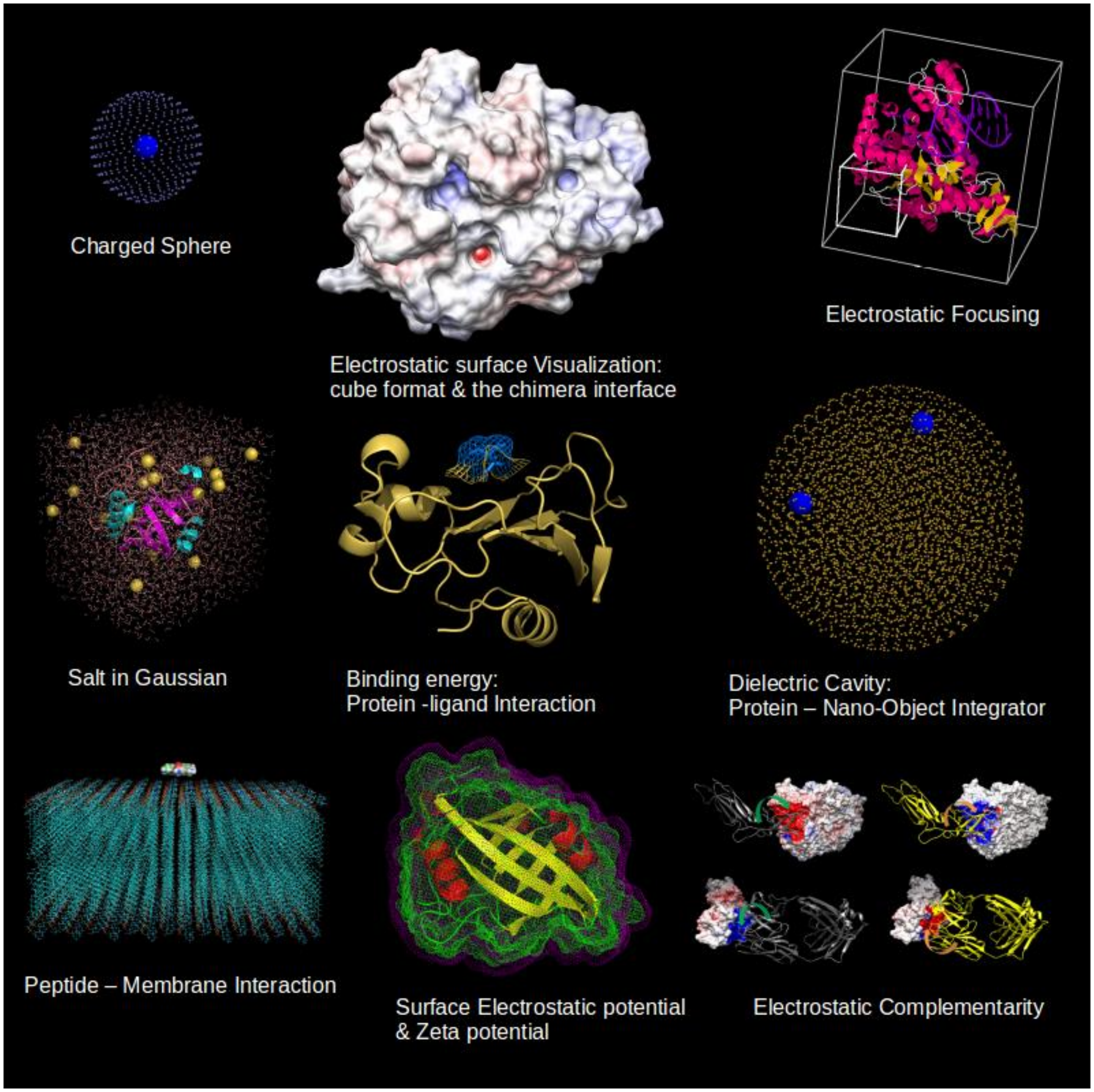

Figure S15. Composite panel illustrating various examples proved within DelPhi distribution.

References:

1. Humphrey, W.; Dalke, A.; Schulten, K., VMD: visual molecular dynamics. *J Mol Graph* **1996,** *14* (1), 33-8, 27-8.
2. Pettersen, E. F.; Goddard, T. D.; Huang, C. C.; Couch, G. S.; Greenblatt, D. M.; Meng, E. C.; Ferrin, T. E., UCSF Chimera--a visualization system for exploratory research and analysis. *J Comput Chem* **2004,** *25* (13), 1605-12.
3. Li, C.; Li, L.; Zhang, J.; Alexov, E., Highly efficient and exact method for parallelization of grid-based algorithms and its implementation in DelPhi. *J Comput Chem* **2012,** *33* (24), 1960-6.

4. Li, C.; Petukh, M.; Li, L.; Alexov, E., Continuous development of schemes for parallel computing of the electrostatics in biological systems: implementation in DelPhi. *J Comput Chem* **2013,** *34* (22), 1949-60.
5. Tanner, D. E.; Chan, K. Y.; Phillips, J. C.; Schulten, K., Parallel Generalized Born Implicit Solvent Calculations with NAMD. *J Chem Theory Comput* **2011,** *7* (11), 3635-3642.
6. Phillips, J. C.; Braun, R.; Wang, W.; Gumbart, J.; Tajkhorshid, E.; Villa, E.; Chipot, C.; Skeel, R. D.; Kale, L.; Schulten, K., Scalable molecular dynamics with NAMD. *J Comput Chem* **2005,** *26* (16), 1781-802.
7. Brooks, B. R.; Brooks, C. L., 3rd; Mackerell, A. D., Jr.; Nilsson, L.; Petrella, R. J.; Roux, B.; Won, Y.; Archontis, G.; Bartels, C.; Boresch, S.; Caflisch, A.; Caves, L.; Cui, Q.; Dinner, A. R.; Feig, M.; Fischer, S.; Gao, J.; Hodoscek, M.; Im, W.; Kuczera, K.; Lazaridis, T.; Ma, J.; Ovchinnikov, V.; Paci, E.; Pastor, R. W.; Post, C. B.; Pu, J. Z.; Schaefer, M.; Tidor, B.; Venable, R. M.; Woodcock, H. L.; Wu, X.; Yang, W.; York, D. M.; Karplus, M., CHARMM: the biomolecular simulation program. *J Comput Chem* **2009,** *30* (10), 1545-614.
8. Li, L.; Li, C.; Sarkar, S.; Zhang, J.; Witham, S.; Zhang, Z.; Wang, L.; Smith, N.; Petukh, M.; Alexov, E., DelPhi: a comprehensive suite for DelPhi software and associated resources. *BMC Biophys* **2012,** *5*, 9.
9. Chakravorty, A.; Jia, Z.; Li, L.; Zhao, S.; Alexov, E., Reproducing the Ensemble Average Polar Solvation Energy of a Protein from a Single Structure: Gaussian-Based Smooth Dielectric Function for Macromolecular Modeling. *J Chem Theory Comput* **2018,** *14* (2), 1020-1032.
10. Chakravorty, A.; Jia, Z.; Peng, Y.; Tajielyato, N.; Wang, L.; Alexov, E., Gaussian-Based Smooth Dielectric Function: A Surface-Free Approach for Modeling Macromolecular Binding in Solvents. *Front Mol Biosci* **2018,** *5*, 25.
11. Jia, Z.; Li, L.; Chakravorty, A.; Alexov, E., Treating ion distribution with Gaussian-based smooth dielectric function in DelPhi. *J Comput Chem* **2017,** *38* (22), 1974-1979.
12. Li, L.; Li, C.; Alexov, E., On the Modeling of Polar Component of Solvation Energy using Smooth Gaussian-Based Dielectric Function. *J Theor Comput Chem* **2014,** *13* (3).
13. Wang, L.; Li, L.; Alexov, E., pKa predictions for proteins, RNAs, and DNAs with the Gaussian dielectric function using DelPhi pKa. *Proteins* **2015,** *83* (12), 2186-97.
14. Chakravorty, A.; Jia, Z.; Li, L.; Alexov, E., A New DelPhi Feature for Modeling Electrostatic Potential around Proteins: Role of Bound Ions and Implications for Zeta-Potential. *Langmuir* **2017,** *33* (9), 2283-2295.
15. Wang, L.; Zhang, M.; Alexov, E., DelPhiPKa web server: predicting pKa of proteins, RNAs and DNAs. *Bioinformatics* **2016,** *32* (4), 614-5.
16. Petukh, M.; Kimmet, T.; Alexov, E., BION web server: predicting non-specifically bound surface ions. *Bioinformatics* **2013,** *29* (6), 805-6.
17. Petukh, M.; Zhang, M.; Alexov, E., Statistical investigation of surface bound ions and further development of BION server to include pH and salt dependence. *J Comput Chem* **2015,** *36* (32), 2381-93.
18. Wang, L.; Li, L.; Alexov, E., pKa predictions for proteins, RNAs, and DNAs with the Gaussian dielectric function using DelPhi pKa. *Proteins: Structure, Function, and Bioinformatics* **2015,** *83* (12), 2186-2197.
19. Wang, L.; Zhang, M.; Alexov, E., DelPhiPKa web server: predicting pKa of proteins, RNAs and DNAs. *Bioinformatics* **2016,** *32* (4), 614-615.
20. Pahari, S.; Sun, L.; Basu, S.; Alexov, E., DelPhiPKa: Including salt in the calculations and enabling polar residues to titrate. *Proteins: Structure, Function, and Bioinformatics* **2018,** *0* (0).
21. Li, L.; Chakravorty, A.; Alexov, E., DelPhiForce, a tool for electrostatic force calculations: Applications to macromolecular binding. *J Comput Chem* **2017,** *38* (9), 584-593.
22. Li, L.; Jia, Z.; Peng, Y.; Chakravorty, A.; Sun, L.; Alexov, E., DelPhiForce web server: electrostatic forces and energy calculations and visualization. *Bioinformatics* **2017,** *33* (22), 3661-3663.

23. Getov, I.; Petukh, M.; Alexov, E., SAAFEC: Predicting the Effect of Single Point Mutations on Protein Folding Free Energy Using a Knowledge-Modified MM/PBSA Approach. *International journal of molecular sciences* **2016,** *17* (4), 512-512.
24. Johnson, D. S.; Mortazavi, A.; Myers, R. M.; Wold, B., Genome-wide mapping of in vivo protein-DNA interactions. *Science* **2007,** *316* (5830), 1497-502.
25. Jimenez, J. S., Protein-DNA interaction at the origin of neurological diseases: a hypothesis. *J Alzheimers Dis* **2010,** *22* (2), 375-91.
26. Robertson, K. D.; Wolffe, A. P., DNA methylation in health and disease. *Nat Rev Genet* **2000,** *1* (1), 11-9.
27. Peng, Y.; Sun, L.; Jia, Z.; Li, L.; Alexov, E., Predicting protein-DNA binding free energy change upon missense mutations using modified MM/PBSA approach: SAMPDI webserver. *Bioinformatics* **2018,** *34* (5), 779-786.
28. Li, L.; Alper, J.; Alexov, E., Multiscale method for modeling binding phenomena involving large objects: application to kinesin motor domains motion along microtubules. *Sci Rep* **2016,** *6*, 23249.
29. Li, L.; Alper, J.; Alexov, E., Cytoplasmic dynein binding, run length, and velocity are guided by long-range electrostatic interactions. *Sci Rep* **2016,** *6*, 31523.
30. Tajielyato, N.; Li, L.; Peng, Y.; Alper, J.; Alexov, E., E-hooks provide guidance and a soft landing for the microtubule binding domain of dynein. *Sci Rep* **2018,** *8* (1), 13266.
